# Dynamical Properties of Postural Control in Obese Community-Dwelling Older Adults †

**DOI:** 10.3390/s18061692

**Published:** 2018-05-24

**Authors:** Christopher W. Frames, Rahul Soangra, Thurmon E. Lockhart, John Lach, Dong Sam Ha, Karen A. Roberto, Abraham Lieberman

**Affiliations:** 1 School of Biological and Health Systems Engineering, Arizona State University, Tempe, AZ 85281, USA; cframes@asu.edu; 2 Muhammad Ali Parkinson Center (MAPC), Barrow Neurological Institute, St. Joseph’s Hospital and Medical Center, Phoenix, AZ 85013, USA; abraham.lieberman@dignityhealth.org; 3 Department of Physical Therapy, Crean College of Health and Behavioral Sciences, Chapman University, Orange, CA 92866, USA; soangra@chapman.edu; 4 Department of Electrical and Computer Engineering, University of Virginia, Charlottesville, VA 22904, USA; jlach@virginia.edu; 5 The Bradley Department of Electrical and Computer Engineering, Virginia Polytechnic Institute and State University, Blacksburg, VA 24061, USA; ha@vt.edu; 6 Center for Gerontology, Virginia Polytechnic Institute and State University, Blacksburg, VA 24061, USA; kroberto@vt.edu

**Keywords:** obesity, postural control, nonlinear

## Abstract

Postural control is a key aspect in preventing falls. The aim of this study was to determine if obesity affected balance in community-dwelling older adults and serve as an indicator of fall risk. The participants were randomly assigned to receive a comprehensive geriatric assessment followed by a longitudinal assessment of their fall history. The standing postural balance was measured for 98 participants with a Body Mass Index (BMI) ranging from 18 to 63 kg/m^2^, using a force plate and an inertial measurement unit affixed at the sternum. Participants’ fall history was recorded over 2 years and participants with at least one fall in the prior year were classified as fallers. The results suggest that body weight/BMI is an additional risk factor for falling in elderly persons and may be an important marker for fall risk. The linear variables of postural analysis suggest that the obese fallers have significantly higher sway area and sway ranges, along with higher root mean square and standard deviation of time series. Additionally, it was found that obese fallers have lower complexity of anterior-posterior center of pressure time series. Future studies should examine more closely the combined effect of aging and obesity on dynamic balance.

## 1. Introduction

Obesity is a growing health problem in older adults [1]. In 2012, approximately 35% of the population above the age of 60 years was considered obese [2]. By 2015, 75% of adults were estimated to be overweight, in which 41% were classified as obese [2,3,4]. Obesity is a complex multifactorial disease associated with risk factors for various diseases and medical complications, including cardiovascular disease [5], atrial fibrillation [6], depression [7,8,9], stroke [8], and a reduction in quality of life [1,2]. Along with the multisystem deterioration that accompanies old age, obesity comports functional decline, sensory deficits [10,11,12,13,14,15,16], and significantly reduced mass-relative lower extremity strength that precipitates falls [17,18]. Compounding the age-related decrements are obesity’s increased mechanical demands that not only increase system constraints, but also prompt an interminable state of physiological and biomechanical compromise: compensatory adaptations to offset the excess trunk mass. It has been reported that obese adults carry an anteriorly displaced center of mass that elicits greater trunk extension while standing in an effort to counteract the excessive weight and maintain balance [19,20,21,22]. As a result, studies show that postural control elicits behavior modifications associated with greater fall risk and injuries [22,23], including increased postural sway area, range, and velocity [19,20,21]. However, not all of literature coincides with these findings, in fact, many studies contradict this and are not explicitly synonymous with fall incidence and injuries [24,25,26], indeed, the literature is rife with contradictions. Some researchers report that obesity necessitates additional balance control constraints that ultimately reduce stability [17,27,28] others report that obesity’s effect on balance is minimal [29,30], merely providing protection from fall-related injuries [24,26].

The ambiguity may be a consequence of the limitations involved in traditional analysis techniques and the lack of consideration for the multifactorial nature of human postural control. Standard variability analysis techniques comprise of linear statistical measures estimating amplitude of center of pressure (COP) excursions. COP displacements equate in linear manner with arbitrary fluctuations in which putative randomness is averaged out, ignoring the time-dependent evolution of the system’s dynamics. A more comprehensive view of postural stability may require the addition of nonlinear measures to characterize the temporal dynamics of the COP time series and evince the underlying motor control processes involved. In this context, the focus of stability is appropriated from the amount of variability in the signal—standard deviation (SD), root mean square (RMS)—to the organization of variability.

To quantify the dynamical properties of postural stability, several nonlinear measures expressed as time series of COP trajectories in both force plate and inertial measurement units (IMU) were employed. Over the last two decades regulatory statistics from nonlinear dynamics, have been used extensively in COP time series analyses to measure neuromuscular connections (feedback) and the subtle changes in postural control [31,32]. By observing the evolution of the postural control system, entropy measures can estimate specific feedback mechanisms and spontaneous properties of interconnected neurons, in which a weak, or degraded neuromuscular system, can be characterized by increased regularity in the physiological time series [33,34]. Furthermore, entropy measures are believed to provide a direct measurement of feedback among neuromuscular connections. Lower entropy indicates high predictability and regularity of time series data whereas high entropy values indicate unpredictability and random variation [33,35].

Weight gain in obesity may alter fractal properties of the motor function. Detrended Fluctuation Analysis (DFA) is a useful technique to characterize the long-range correlations of a time series and provides complementary insight and also reveals the underlying complexity into the multifactorial nature of human postural control. The present study utilizes, both forceplates and inertial sensors to evaluate postural sway which is characterized by sway area (elliptical and circular area) and mean power frequency (MPF), standard deviation (SD), root mean square (RMS), range, mean COP velocity and path length of COP signals. However, an increase in body mass may induce subtle impairments in balance and without obviously detectable unsteadiness.

This study is an extended version of our previous study [3] and has explored the responsiveness of linear and nonlinear postural measures to evaluate the effects of increased body weight on postural stability and fall risk in obese community-dwelling elderly adults.

## 2. Materials and Methods

Ninety-eight community-dwelling older adults participated in the study. Demographics of population are provided in Table 1, anthropometric information in Table 2 and gender ratio in Table 3. This sample size was selected to provide smaller confidence interval on the estimated error rate when classifying fallers and non-fallers [36]. Study participants were divided into three groups based on their BMI: normal (19 ≤ BMI < 25 kg/m^2^), overweight (25 ≤ BMI < 30 kg/m^2^), and obese (BMI ≥ 30 kg/m^2^) [36]. Fall history was recorded, retrospectively, for 2-years with emphasis on fall frequency and characteristics of the falls. Any person with at least one fall in the prior year was classified as a faller and the others as non-fallers; demographics of participants is shown in Table 1, Table 2 and Table 3. The study was conducted in four separate senior community centers throughout Virginia, using the IMU and force plate on four different days. This study was approved by the Virginia Tech Institutional Review Board (VT-IRB) and was conducted in collaboration with Northern Virginia Fall Prevention Coalition (NVFPC) and INOVA Hospital. All participants provided written consent which was approved by VT_IRB prior to their participation. All measurements were performed barefoot in quiet standing, looking in the forward direction, with their foot placement standardized. For postural stability, the participants were asked to stand in two visual conditions: eyes open (EO) and eyes closed (EC). Each measurement lasted for 60 s and was repeated twice. The sampling rate inertial sensors and forceplate was 100 Hz. A rest of 3 min was provided between each measurement. For the analysis, the COP trajectory was separated into its mediolateral, ML and anteroposterior, AP, components. BMI was calculated for each participant based on his/her height and weight. The recorded COP signals were filtered using a fourth-order low-pass Butterworth filter, with a cut-off frequency of 5 Hz to eliminate measurement noise. Given the limited data length, measurements began a few seconds after the informed start of the trial and ended a few seconds before the informed termination of the trial. All analysis was performed using custom Matlab routines (The Mathworks, Version 2015a). A mixed effect MANOVA model was used, with participants being the random effect. Because the design was unbalanced, we used restricted maximum likelihood (REML) as the fitting method using JMP (JMP^®^, Pro 10.0.2. SAS Institute Inc., Cary, NC, USA, 1989–2007).

Methods for entropy and detrended fluctuation analysis are provided below similar to our previous study [3].

We hereby provide information pertaining to non-linear analysis performed similar to our previous work [3]. Approximate entropy (ApEn) quantifies the ensemble amount of randomness, or irregularity [37]. Here in this study, we employ ApEn as measure of complexity to quantify COP time series based non-linear variability during quiet standing in community-dwelling older adults. Some of the earlier research has reported that ApEn is sensitive enough too and can detect subtle changes in COP variability which may not be apparent in traditional biomechanical measures of postural stability [31,38], such as COP area, sway velocity, path length etc. The concept of Approximate Entropy (ApEn) was firstly reported by Pincus [39]. Although ApEn can be computed for any timeseries, here, we explain the approach of ApEn estimation as applied to center of pressure (COP) timeseries data. ApEn works on logarithmic likelihood such that the patterns of the nearby data have similar pattern. For example a sequence of total N numbers of COP time series e.g., COPx(1), COPx(2),…, COPx(N). To compute ApEn, m-dimensional vector sequences *p_m_* (*i*) were constructed from the COP time series like [*p_m_* (1), *p_m_* (2),…, *p_m_* (N − m + 1)], where the index *i* can take values ranging from 1 to N – m + 1. Where the distance between two vectors *p_m_* (*i*) and *p_m_* (*j*) is defined as |*p_m_* (*j*) − *p_m_* (*i*)|,

(1) $$C_{i}^{m}\left(d\right)=\frac{1}{N-m+1}\;\mathrm{such}\;\mathrm{that}\;\left|P_{m}\left(j\right)-P_{m}\left(i\right)\right|<d$$

where m is the pattern length selected as 2, *d* is the similarity coefficient which has been set to 0.2% of the standard deviation of total length of COP data [33]. These constants have previously yielded statistically reliable and reproducible results. $C_{i}^{m}\left(d\right)$ is considered as the mean of the fraction of patterns of length *m* that resemble the pattern of the same length that begins at index *i*. ApEn is computed as:

(2) $$\mathrm{ApEn}\left(N,m,d\right)={\left(N-m+1\right)}^{-1}\overset{N-\left(m-1\right)}{\underset{i=1}{\sum }}{\mathrm{lnC}}_{i}^{m}\left(d\right)-{\left(N-m\right)}^{-1}\overset{N-m}{\underset{i=1}{\sum }}{\mathrm{lnC}}_{i}^{m+1}\left(d\right)$$

ApEn is a unitless value between 0 and 2 [37]. Smaller ApEn values indicate a higher probability of regular repeating sequences and less complex timeseries. An ApEn value of zero, depicts that the time series is perfectly repeatable (for example periodic sinewave), whereas, the value of 2 is produced by random time series, for which repeating sequences only occur by chance (example Gaussian noise). Thus, the input parameters for the ApEn calculation were (1) a pattern length (m) of 2 data points; (2) a tolerance window normalized to 0.2 times the standard deviation of individual time series. The pattern length (*m*) and tolerance level (*r*) were chosen as per previous research using COP [31,32,40].

Signal regularity was also quantified using sample entropy (SaEn). SaEn indexes the regularity of a time series by calculating the probability that having a repeated signal for a window length *m*, will remain similar for *m* + 1 data points—excluding any self-matches and within a matching tolerance *r*. The greater SaEn values delineate irregularity and rate generation of new information, in which a set of similar points are considered unique as they will likely not be followed by a similar set of matching points within a specified tolerance *r*. Higher SaEn values are considered part of a healthy, robust system able to adapt to challenges and unexpected perturbations. Lower values are associated with greater regularity of the time series, in which there is a greater likelihood that sets of matching epochs in a time series will be followed by another match within a specified tolerance *r*. Lower values denote a possible rigid, disease state unable to adapt to challenges. For the present study, SaEn was computed with the COP time series and the increment of the COP time series in both the AP and ML directions. Parameters *m* and *r* were chosen according to the procedure described by Lake et al. Ramdani et al. (2011) obtaining *m* = 3 and *r* = 0.25 for both directions [6,41,42].

(3) $$\mathrm{SaEn}\left(N,m,r\right)=\ln\left[\frac{\sum_{i=1}^{N-\left(m-1\right)}C_{i}^{m}\left(r\right)}{\sum_{i=1}^{N-m}C_{i}^{m+1}\left(r\right)}\right]$$

**Figure 1:**
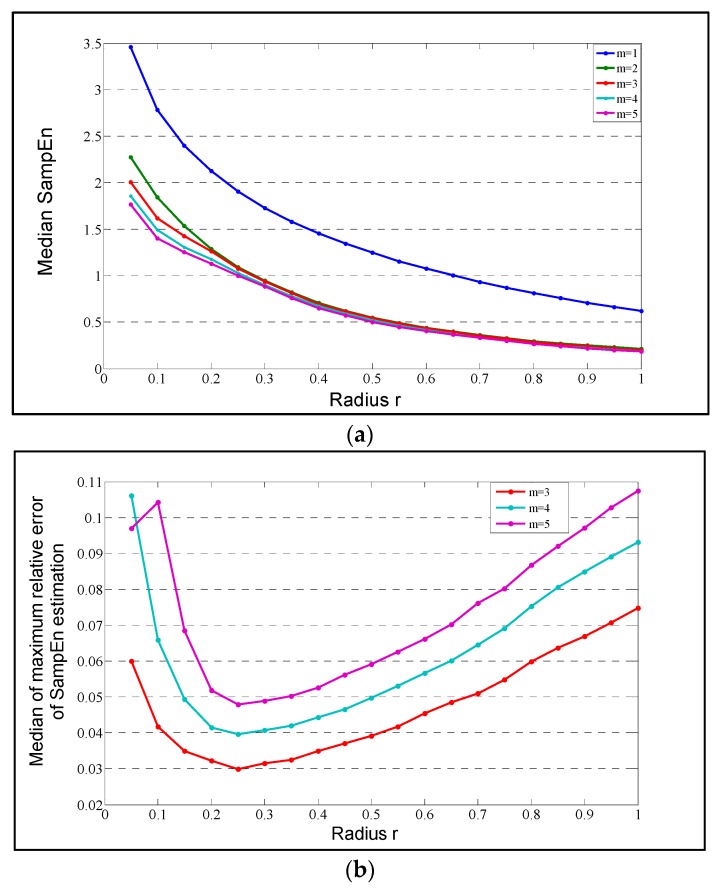
(**a**) Median over all the center of pressure (COP) AP time series; (**b**) The lowest curve is obtained for *m* = 3 as it shows a minimum that is lower than 0.05. This minimum is reached for *r* = 0.25 for both AP and ML directions.

Multiscale entropy (MSE) is a regularity measure that quantifies the information content of postural fluctuations over a range of physiologically relevant time scales while sample entropy is computed for every consecutive coarse-grained time series. The entropy values are then plotted as a function of the time scales in which the area under the curve reveals the signal’s complexity index (CI). A complex signal is associated with a time evolution with a rich structure on multiple scales. For white noise, which is irregular on small time scales but not structurally complex, the entropy decreases for larger time scales. For a complex signal, such as pink 1/f noise, the entropy remains high on different scales. For the computation of MSE the input parameters *m* = 3 and *r* = 0.25 were chosen similar to the SaEn algorithm as shown in Figure 1b.

Detrended fluctuation analysis (DFA) is a nonlinear analysis tool used to detect long range correlations in time series with nonstationarity [43]. Firstly, intrinsic trends are removed as trends could mislead for long range correlations. DFA provides insights into scaling behavior of natural variability in time series. The COP time series are also non-stationary [44,45]. Time series data is systematically divided into segments of different lengths (scales). Fluctuation analysis is then performed as sum of the residuals squared divided by segment length. Finally, a log-log plot of the average error (fluctuation) versus segment length (scale) is performed. The slope of this plot is the scaling exponent α (DFA parameter). Pure random walk has α as 1.5 and white noise α is 0.5 [46]. DFA is computed in two steps:

The time series *B*(*k*) is shifted by the mean <*B*> and integrated (cumulatively summed),

(4) $$y\left(k\right)=\overset{k}{\underset{i=1}{\sum }}\left[B(i\right)-<B>]$$

Then segmented into windows of various sizes Δ*n*

In each segmentation the integrated data is locally fit to a polynomial *y*_Δ*n*_(*k*) and mean-squared residual *F*(Δ*n*) (fluctuations) with N as total number of data points

(5) $$F\left(\Delta n\right)=\sqrt{\frac{1}{N}}\overset{N}{\underset{k=1}{\sum }}{\left[y\left(k\right)-y_{\Delta n}\left(k\right)\right]}^{2}$$

*F*^2^(Δ*n*) is the average of the summed squares of the residual in windows. DFA procedure tests for self-similarity or fractal properties at different resolutions (windows sizes).

(6) $$F\left(\Delta n\right)=C{\left(\Delta n\right)}^{\propto }$$

where *C* is a constant and α is estimated from a least-square fit.

(7) ln(F(Δn))=⋉ln(Δn)+ln(C)

This scaling coefficient α is a measure of correlation in the noise and an estimate of the Hurst exponent *H*.

The median of the maximum relative error *Q*(*m*,*r*) of the *SaEn* calculation as a function of *r* = 0.25 and *m* = 3.

## 3. Results

### 3.1. Linear Measures

**Figure 2:**
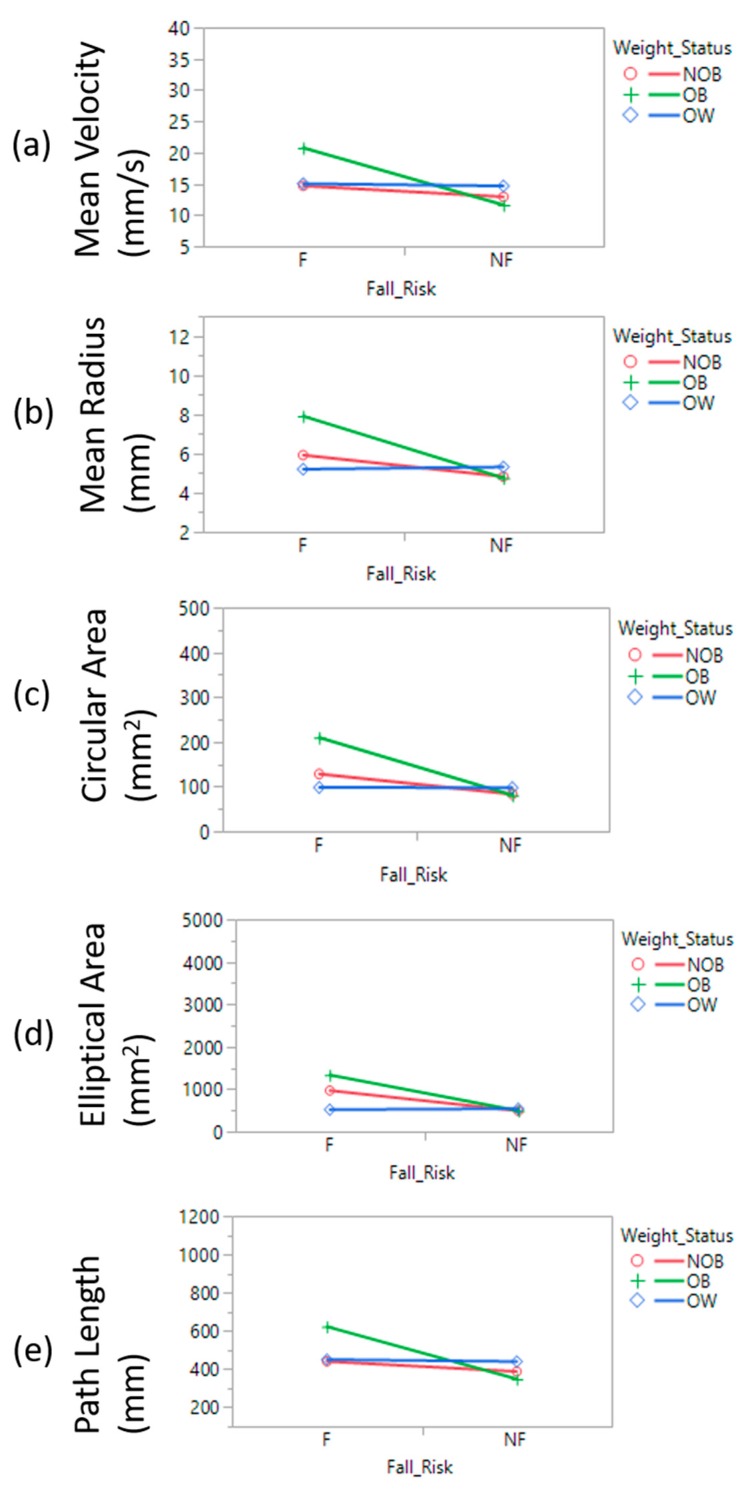
Linear measures of postural stability (**a**) Mean Velocity; (**b**) Mean Radius; (**c**) Circular area; (**d**) Elliptical area; (**e**) COP Path length.

Significant differences were observed in a multitude of linear force plate measures comparing obese fallers and obese non-fallers: Sway area (95% confidence ellipse, *p* = 0.0008, *F* = 7.39; circular area, *p* < 0.0001, *F* = 9.80), mean velocity (*p* = 0.001, *F* = 6.51), and mean path length of COP (*p* = 0.001, *F* = 6.51); the eyes-open (EO) vs eyes-closed (EO) condition afforded similar results (Figure 2). Obese fallers demonstrated significantly higher sway range (*p*-value = 0.001, *F* = 7.44), RMS values (*p*-value = 0.002, *F* = 6.62) and SD values (*p*-value = 0.002, *F* = 6.62) from the force plate COP time series. Significant statistical variability between obese fallers and obese non-fallers was similarly observed utilizing the IMU: Sway area (ellipse area, *p* = 0.003, *F* = 5.89; and circular area *p* < 0.0002, *F* = 8.97), mean velocity (*p* = 0.011, *F* = 4.56), mean radius (*p* < 0.0001, *F* = 10.47) (Table 4) and mean path length of COP (*p* = 0.011, *F* = 4.56). Further traditional postural stability parameters with eyes open and eyes closed condition are shown in Table 5.

Similarly, sway range (*p*-value = 0.002, *F* = 6.22), RMS-value (*p*-value = 0.001, *F* = 7.19) and SD-values (*p*-value = 0.004, *F* = 5.86) were significantly higher in obese fallers from IMU time series. Mean power frequency (MPF) of the time series in eyes closed condition were found to be significantly higher than in eyes open condition (*p* value < 0.0001, *F* = 23.89) for all elderly participants.

### 3.2. Nonlinear Measures

**Figure 3:**
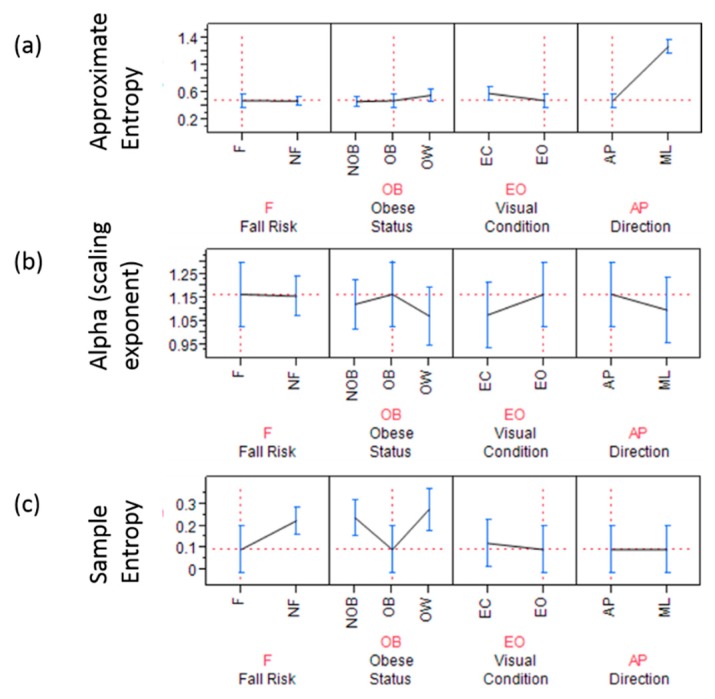
Forceplate signal based non-linear analysis showing (**a**) approximate entropy; the graph highlights (dashed red line) obese fallers have significantly lower complexity in anterior posterior direction with eyes open condition; (**b**) scaling exponent α and; the graph highlights (dashed red line) that obese fallers have significantly higher scaling exponents in anterior posterior direction during eyes open condition (**c**) sample entropy is significantly lower for AP time series derived from forceplate for fallers with obesity in eyes open condition (red dashed lines).

The α scaling exponent from DFA utilizing both the force plate and IMU signals, did not reach significance for any of the fall and obese conditions, respectively. However, the general trend was that in the eyes open condition, α was higher than in the eyes closed condition. Anterior-posterior COP times series were found to have significantly higher persistence than in mediolateral direction time series (Figure 3). It was also seen (Table 6) that obese fallers had higher persistence than non-obese and overweight.

Regarding COP fluctuations taken from the force plate, approximate entropy (*p* < 0.0001, *F* = 2957.9) in the AP direction was significantly lower than in the ML direction in obese as well as in non-obese and overweight older adults (Figure 3). Whereas, scaling exponent (alpha) (*p*-value = 0.03, *F* = 4.75) in the AP direction was significantly higher than ML direction in obese as well as in non-obese and overweight elderly persons. Sample entropy in the AP direction during the eyes open condition was found to be significantly lower in obese fallers (*p* = 0.007, *F* = 4.95) than other non-obese and overweight elderly persons (Figure 3).

**Figure 4:**
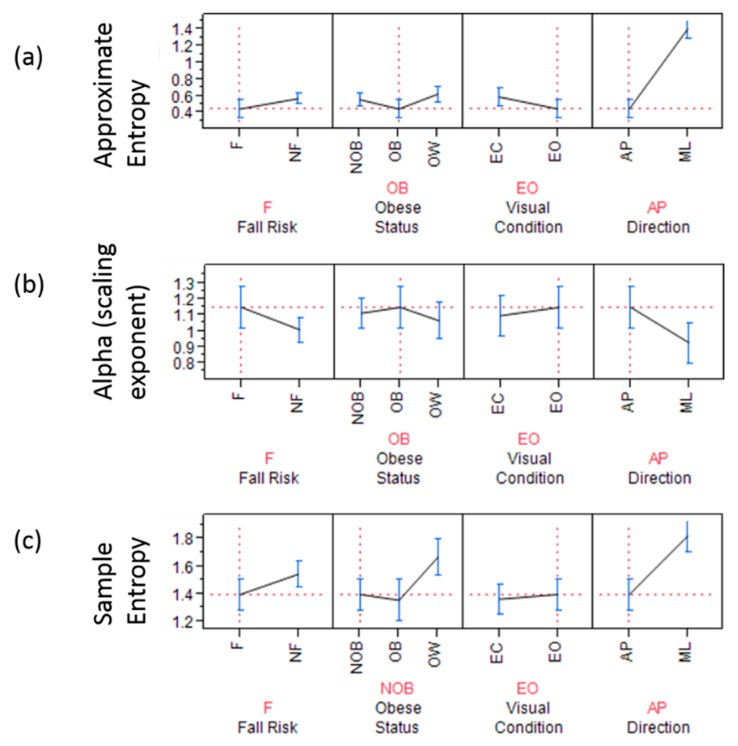
Inertial measurement units (IMU) based nonlinear analysis showing (**a**) approximate entropy; The graph highlights (dashed red-line) approximate entropy is significantly lower in obese fallers in anterior posterior direction during eyes open double limb stance; (**b**) scaling exponent α and; The graph highlights (dashed red-line) that the scaling exponent is significantly higher for obese fallers in anterior posterior direction during eyes open double limb stance; (**c**) sample entropy; The graph highlights (red dashed line) that fallers who were non-obese showed significantly lower complexity (measured by sample entropy)for AP time series derived from IMU.

**Figure 5:**
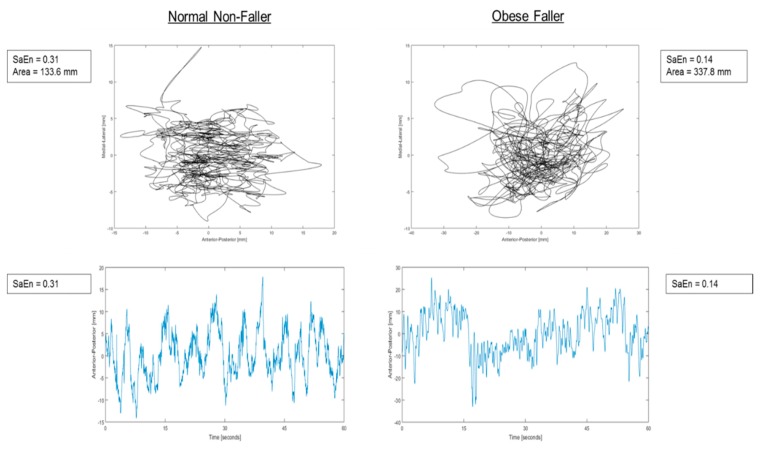
Ensemble patterns of postural stability of fallers and non-fallers exhibiting fallers with larger area of sway with lower sample entropy.

**Figure 6:**
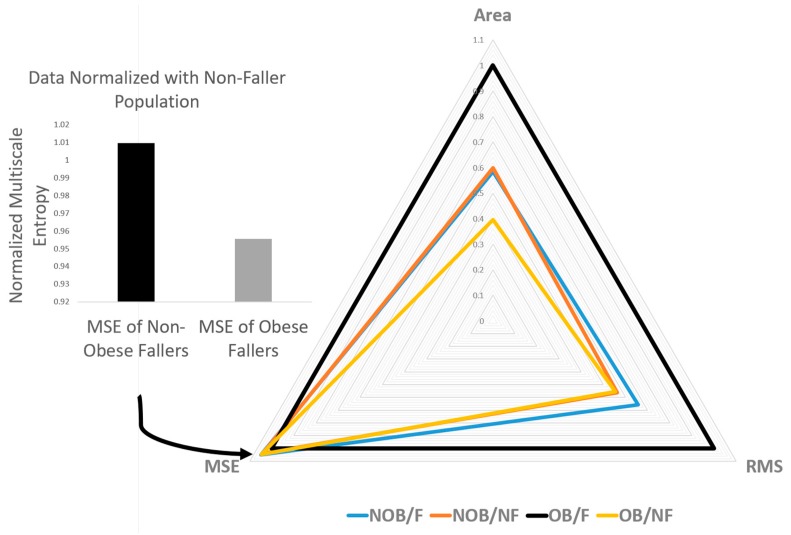
Radar plot of significant discriminative parameters.

COP signals from the IMU revealed that approximate entropy (*p* < 0.0001, *F* = 2857.7) in the AP direction was significantly lower than in the ML direction in the obese participants as well as in the non-obese and overweight elderly individuals (Figure 4). Whereas, the scaling exponent (alpha) (*p*-value < 0.0001, 54.37) in the AP direction was significantly higher than ML direction in obese as well as in non-obese and overweight elderly individuals. Sample entropy in AP direction was found to be significantly lower in obese fallers (*p* = 0.015, *F* = 4.21) than other non-obese and overweight elderly individuals (Figure 4 and Figure 5). Figure 6 shows discriminative parameters for obesity.

## 4. Discussion

The present study investigated the effects of obesity on fall risk in community-dwelling older adults, utilizing nonlinear analyses on signals acquired from force plate and IMU measurements. It was hypothesized that body weight-related factors increased fall risk in obese older adults identified by linear and nonlinear measures of postural sway. A significant increase in linear parameters (mean radius, ellipse area, sway range, RMS, SD) was identified for obese older adults. Nonlinear regularity measures through sample entropy revealed that the presence of obesity and fall risk had loss of complexity (lower sample entropy values) in eyes open condition in AP sway signals (Figure 3c and Figure 6). It was also found that obese fallers (Table 6) had higher persistence than non-obese and overweight older adults. Complexity in the ML direction of COP time series was significantly higher in obese participants than that in non-obese and overweight community-dwelling elderly people [46,47,48].

Statistical variability, such as range and standard deviation, reflect the overall magnitude of COP displacement without considering the temporal structure of COP time series. However, nonlinear measures of postural signals reveal subtle temporal properties of signals which are not detected in obese individuals through a traditional linear approach [27,28,31]. Traditionally, greater COP displacements in anterior posterior and medial-lateral directions have been linked with less stability and consequently, pathology [49]. Although implicated, as the biological systems are intrinsically complex and the linear analysis alone may not account for the time-dependent evolution of the complex system hidden within the time series of COP displacements. As such, an increased excursion of COP may not be an indicator of deficient postural control system, rather, it may be a healthy, vigilant adaptable system capable of adapting to unexpected perturbations for balance maintenance.

In the present study, entropy-based estimations of organizational variability delineate the adaptive capacity of obese participants to maintain balance (lower ApEn and SaEn values indicate greater regularity and decreased complexity). These results are in agreement with previous studies linking aging and pathology [3,47]. It was also found that movements were constrained in the AP direction compared to the ML direction leading to less complex, more stable response modes—a more regular sway pattern with closed-loop short term dependencies to restore balance. Hence, the motor system is probably unable to adjust to the demands inherent to obesity and overweight characteristics, therefore movements transition to a more rigid postural control behavior (repeated patterns and decreased complexity) in the AP direction that diminish both adaptability and stability. In essence, the increase in regularity and possible decrease in complexity may be a result of impaired feedback control or impaired proprioception [50] leading to a reduced adaptive capacity of the postural system [48]. Obese or overweight individuals make hyper activation of plantar mechanoreceptors due to continuous pressure of supporting a large mass, which leads to reduced plantar sensitivity [28,51]. Moreover, the firing of postural muscles may follow an adaptive strategy to reduce joint loads in obese elderly persons that diminish postural stability. Fractal analysis of the COP time series revealed relatively marginal differences in obese fallers versus non-obese and overweight fallers in both the AP and ML directions which were not found to be statistically significant. Obese fallers generally had higher α values in the eyes-open condition (1.23 vs. 1.22) relative to eyes-closed conditions, without reaching significance. From a biomechanics perspective, it may also be due to inability of elderly people to control and accelerate center of mass (COM) over base of support, perhaps due to lack of strength and degradation of type II fibers in skeletal muscles. While muscle strength was not objectively measured in this study, it has been documented that many older people have relatively weaker tibialis anterior and vastus lateralis muscle strength compared to that of healthy adults [52,53]. Obesity is also found related with lower level of physical activity and impaired cardiorespiratory fitness and knee strength compared to lean counterparts [54], possibly impairing obese persons’ ability to correct a shift in the body’s center of mass and effectively prevent from falling. Probably an increased postural sway could be an adaptive strategy in obese individuals to provide additional stability under conditions of weakness in muscles involved for postural control. Age-related deterioration of sensory and neuromuscular control mechanisms could have definitely added to this problem. Degradation of balance shows that fall risk is increased in those with higher BMI.

Obese elderly persons adopt compensatory strategies, despite their report of having no difficulty in performing the same task as lean counterparts [55]. We assume several mechanisms might have accounted for poor postural balance in obese older adults. First, as body mass of various segments increases, the energy and the strength required to bring the COM over the base of support increases correspondingly similar to when ambulating [56,57]. This may lead to extra biomechanical burden in lower extremity joints to maintain balance, thus obese elderly individuals are liable to adopt an adaptive strategy during quiet standing (perhaps a more closely posture or rigid fixed system with reduction in system degrees of freedom). Secondly, undoubtedly aging is associated with progressive muscle loss (specifically Type II fiber) and which could have resulted in muscular weakness and fat infiltration [58].

These methods build on narrative descriptions of variability in obesity-related postural control by quantifying qualities of postural control, such as complexity. Complexity can be described by the regularity of the pattern of variability and by the number of strategies used over time. In combination, linear and nonlinear analysis quantify postural control to provide a more complete understanding of the adaptive strategies used in postural control than either method could provide alone. The strength of the conclusions of this study must be tempered by the study’s limitations. The older participants were aware that they were participating in a fall risk assessment protocol. This could be a bias in the population studied. They may be conscious of the environment and their performance may have been affected by the environment. We tested balance of community-dwelling older adults in four different community centers, and the environment of data collection may also have been a confound in this study. The community center setting in which data were obtained for this study provided a familiar environment for the older participants. At the same time, the non-laboratory setting limited the scope of this data. Howsoever, such analyses may provide insight as to the potential fall risk associated with elderly obese participants.

## 5. Conclusions

Obesity in older adults is recognized as an important issue with fall risk implications. However, little is known about the relationship between obese elderly persons and their gait characteristics. With fractal analysis, we have not found differences between the results from faller/non-faller and obese/non-obese/overweight groups under EO and EC conditions using both the instruments force plate COP and IMU COP. This indicates that DFA is not able to elucidate the role played by body weight and faller/on-faller status. Although α was found to be higher for the AP direction and for the EO condition, which shows that COP trajectories are more persistence in AP direction and in EO condition (Table 6). With obesity, ApEn revealed a change in the randomness of COP oscillations that occurred in eyes open (EO) visual condition in anterior-posterior direction. Obese elderly persons were found to have significantly lower randomness in the AP direction (or lower entropy) (*p* < 0.0001, *F* = 2957.9).

The present study suggests that the body-weight influences postural balance in obese elderly individuals and both traditional biomechanical parameters as well as non-linear measures could help detect fall risk in persons who are obese. Our results are consistent with recent findings by Rossi-Izquierdo et al. [59]. Inertial sensors can be used to detect fall risk caused by higher body mass in elderly individuals. Indeed, our findings indicate that a change in temporal structure of COP variability as seen by ApEn and SaEn can detect postural changes due to obesity in elderly persons and IMUs may serve as alternative instrument in assessing this. Although implicated, further studies are warranted to elucidate the dynamics of fall recovery to provide comprehensive interpretations of fall risks in the aging population.

## Figures and Tables

**Table 1:** Demographics of the subject population.

Health Status	Faller (F)	Non-Faller (NF)
Non-obese (NOB)	14	20
Obese (OB)	8	22
Overweight (OW)	10	24

**Table 2:** Age, height, weight and BMI ratio of each group.

	Fall Risk			
	Faller		Non-Faller	
	NOB	OB	NOB	OB
Age (years)	76.82 ± 6.87	72.29 ± 4.72	77.41 ± 8.49	72.68 ± 7.40
Height (m)	1.71 ± 0.06	1.61 ± 0.07	1.67 ± 0.11	1.64 ± 0.05
Weight (kg)	79.14 ± 8.18	80.77 ± 21.98	67.26 ± 12.41	87.66 ± 21.05
BMI (kg/m^2^)	26.85 ± 2.08	31.27 ± 8.09	24.29 ± 2.16	32.65 ± 7.62

**Table 3:** Gender ratio for each group.

Gender	Fall Risk		Weight Status		
	F	NF	NOB	OB	OW
Female	12	53	21	19	25
Male	6	27	13	11	9

**Table 4:** Means and standard deviations of force plate parameters.

	NOB		OB		OW	
	F	NF	F	NF	F	NF
**Area ^1^**	9.69 ± 1.20	4.91 ± 3.57	13.36 ± 7.30	4.92 ± 3.90	5.19 ± 4.96	5.41 ± 3.52
**Range AP ^2^**	34.29 ± 14.44	26.53 ± 8.68	51.78 ± 20.77	26.73 ± 10.48	29.24 ± 13.47	30.49 ± 7.58
**Range ML ^2^**	15.40 ± 10.66	11.31 ± 5.36	17.42 ± 4.12	11.41 ± 6.39	11.31 ± 8.06	11.18 ± 5.54
**RMS AP**	6.20 ± 2.25	5.13 ± 1.87	8.87 ± 3.33	5.18 ± 2.06	5.60 ± 2.32	5.93 ± 1.82
**RMS ML**	3.05 ± 1.95	2.18 ± 1.15	3.53 ± 0.86	2.10 ± 1.21	2.15 ± 1.63	2.06 ± 0.94
**SD AP**	6.20 ± 2.25	5.13 ± 1.87	8.87 ± 3.33	5.19 ± 2.06	5.60 ± 2.32	5.94 ± 1.82
**SD ML**	3.05 ± 1.95	2.18 ± 1.15	3.53 ± 0.86	2.10 ± 1.21	2.16 ± 1.63	2.07 ± 0.94
**MPF AP**	0.36 ± 0.05	0.38 ± 0.06	0.38 ± 0.05	0.37 ± 0.05	0.40 ± 0.05	0.40 ± 0.06
**MPF ML**	0.39 ± 0.06	0.40 ± 0.07	0.38 ± 0.07	0.39 ± 0.06	0.42 ± 0.08	0.40 ± 0.06
**DFA α AP**	1.12 ± 0.16	1.14 ± 0.16	1.16 ± 0.23	1.15 ± 0.18	1.07 ± 0.15	1.09 ± 0.16
**DFA α ML**	1.37 ± 0.13	1.33 ± 0.12	1.40 ± 0.09	1.37 ± 0.10	1.30 ± 0.15	1.35 ± 0.12
**ApEn AP**	0.47 ± 0.12	0.54 ± 0.18	0.48 ± 0.14	0.48 ± 0.14	0.56 ± 0.12	0.52 ± 0.17
**ApEn ML**	0.55 ± 0.23	0.57 ± 0.23	0.44 ± 0.13	0.60 ± 0.26	0.62 ± 0.17	0.57 ± 0.21
**SaEn AP**	0.23 ± 0.22	0.25 ± 0.21	0.09 ± 0.03	0.22 ± 0.18	0.28 ± 0.21	0.19 ± 0.14
**SaEn ML**	0.23 ± 0.22	0.25 ± 0.21	0.09 ± 0.03	0.22 ± 0.18	0.28 ± 0.20	0.19 ± 0.14
**MSE AP**	3.41 ± 1.03	4.13 ± 1.51	3.73 ± 1.68	3.33 ± 1.13	4.18 ± 0.98	4.09 ± 1.52
**MSE ML**	3.41 ± 1.30	3.24 ± 1.03	3.17 ± 1.17	3.42 ± 1.51	3.58 ± 1.16	3.68 ± 1.27

^1^ 95% confidence ellipse area (cm^2^); ^2^ units in cm.

**Table 5:** Mean and standard deviations of forceplate measures during Eyes Open and Eyes Closed conditions.

	Weight Status					
	NOB		OB		OW	
	Eyes Closed	Eyes Open	Eyes Closed	Eyes Open	Eyes Closed	Eyes Open
**MPF**	0.42 ± 0.07	0.39 ± 0.06	0.42 ± 0.08	0.38 ± 0.06	0.44 ± 0.07	0.41 ± 0.07
**RMS**	4.54 ± 2.65	4.06 ± 2.38	5.12 ± 3.93	4.33 ± 2.91	4.51 ± 2.51	3.97 ± 2.47
**Range**	24.66 ± 15.03	21.36 ± 13.14	26.08 ± 18.76	23.22 ± 16.69	24.00 ± 13.69	20.68 ± 12.42
**DFA**	0.99 ± 0.19	1.06 ± 0.21	1.04 ± 0.23	1.09 ± 0.21	0.98 ± 0.19	1.05 ± 0.18
**ApEn**	0.63 ± 0.21	0.54 ± 0.20	0.58 ± 0.18	0.52 ± 0.20	0.63 ± 0.17	0.56 ± 0.18
**MSE**	4.34 ± 1.74	3.58 ± 1.28	4.20 ± 1.58	3.40 ± 1.34	4.55 ± 1.59	3.89 ± 1.31

**Table 6:** Means and standard deviations of IMU parameters.

	NOB		OB		OW	
	F	NF	F	NF	F	NF
**Area ^1^**	40.17 ± 34.11	41.24 ± 43.69	68.87 ± 20.91	27.20 ± 14.16	32.83 ± 21.30	27.36 ± 15.74
**Range AP ^2^**	0.36 ± 0.05	0.36 ± 0.04	0.37 ± 0.07	0.36 ± 0.04	0.38 ± 0.05	0.36 ± 0.05
**Range ML ^2^**	1.58 ± 0.92	1.32 ± 0.44	2.17 ± 0.66	1.38 ± 0.50	1.16 ± 0.37	1.43 ± 0.55
**RMS AP**	1.74 ± 1.01	1.41 ± 0.42	2.49 ± 0.57	1.47 ± 0.57	1.23 ± 0.38	1.63 ± 0.62
**RMS ML**	8.19 ± 5.19	7.01 ± 2.26	12.45 ± 2.75	6.89 ± 2.17	6.41 ± 2.30	7.06 ± 2.19
**SD AP**	1.11 ± 0.21	1.11 ± 0.16	1.15 ± 0.22	1.01 ± 0.19	1.06 ± 0.12	1.05 ± 0.18
**SD ML**	1.26 ± 0.10	1.29 ± 0.12	1.27 ± 0.09	1.31 ± 0.09	1.36 ± 0.02	1.27 ± 0.09
**MPF AP**	1.40 ± 0.31	1.54 ± 0.27	1.35 ± 0.19	1.54 ± 0.21	1.67 ± 0.11	1.47 ± 0.21
**MPF ML**	8.43 ± 2.28	9.23 ± 2.25	7.84 ± 1.96	9.20 ± 2.04	10.71 ± 1.40	8.21 ± 2.28
**DFA α AP**	0.44 ± 0.07	0.41 ± 0.05	0.43 ± 0.04	0.41 ± 0.05	0.44 ± 0.07	0.42 ± 0.06
**DFA α ML**	0.49 ± 0.21	0.55 ± 0.32	0.62 ± 0.15	0.44 ± 0.13	0.54 ± 0.34	0.42 ± 0.13
**ApEn AP**	0.56 ± 0.28	0.59 ± 0.33	0.75 ± 0.18	0.52 ± 0.18	0.61 ± 0.34	0.47 ± 0.14
**ApEn ML**	3.12 ± 1.52	3.93 ± 3.50	3.87 ± 0.70	2.51 ± 0.64	3.57 ± 2.41	2.62 ± 1.09
**SaEn AP**	0.96 ± 0.25	0.95 ± 0.14	0.93 ± 0.22	0.95 ± 0.15	0.88 ± 0.24	0.97 ± 0.16
**SaEn ML**	1.40 ± 0.04	1.39 ± 0.04	1.41 ± 0.04	1.42 ± 0.03	1.39 ± 0.08	1.41 ± 0.03
**MSE AP**	1.82 ± 0.15	1.82 ± 0.14	1.82 ± 0.12	1.90 ± 0.10	1.86 ± 0.21	1.88 ± 0.09
**MSE ML**	12.60 ± 2.00	12.48 ± 1.71	12.01 ± 1.32	12.57 ± 1.36	12.64 ± 1.97	12.78 ± 1.28

^1^ 95% confidence ellipse area (cm^2^); ^2^ units in cm.
